# High-Content, Image-Based Screening for Drug Targets in Yeast

**DOI:** 10.1371/journal.pone.0010177

**Published:** 2010-04-14

**Authors:** Shinsuke Ohnuki, Satomi Oka, Satoru Nogami, Yoshikazu Ohya

**Affiliations:** Department of Integrated Biosciences, Graduate School of Frontier Sciences, The University of Tokyo, Kashiwa, Chiba, Japan; Centre for Genomic Regulation, Spain

## Abstract

**Background:**

Drug discovery and development are predicated on elucidation of the potential mechanisms of action and cellular targets of candidate chemical compounds. Recent advances in high-content imaging techniques allow simultaneous analysis of a range of cellular events. In this study, we propose a novel strategy to identify drug targets by combining genetic screening and high-content imaging in yeast.

**Methodology:**

In this approach, we infer the cellular functions affected by candidate drugs by comparing morphologic changes induced by the compounds with the phenotypes of yeast mutants.

**Conclusions:**

Using this method and four well-characterized reagents, we successfully identified previously known target genes of the compounds as well as other genes involved with functionally related cellular pathways. This is the first demonstration of a genetic high-content assay that can be used to identify drug targets based on morphologic phenotypes of a reference mutant panel.

## Introduction

Medications exert their pharmacologic effects by interacting with a wide range of cellular components. To facilitate drug discovery and development, methods are needed to identify cellular targets and elucidate the mechanisms of action of candidate chemical compounds. Conventional drug screening approaches that focus on specific biochemical activities allow the identification of compounds that target the particular activities, but the selected compounds often have multiple *in vivo* targets that must be identified. Alternative approaches involve cell-based screens that account for interactions within the whole cell; however, *in vivo* targets must still be identified because cell-based screens focus on the desired cellular response rather than the biomolecular activity of the targets.

A recent study in *Saccharomyces cerevisiae* used a comprehensive panel of yeast deletion mutants and microarray technology to facilitate the identification of the intracellular targets of a compound [1]. For example, mutants that show a specific sensitivity or resistance to a candidate drug can be selected from the yeast mutant pool using a fitness-based approach combined with a yeast DNA barcode array [2], [3], [4]. Alternatively, a compendium approach examining multiple cellular response parameters (*e.g.*, gene expression levels and growth rates) can be used to infer the drug targets of a novel compound based on reference bioactivity profiles of well-characterized drugs [5], [6].

Fluorescence microscopic imaging is advantageous for high-content assays that assess *in vivo* drug effects using multiple cellular response parameters [7]. To examine a number of intracellular events in *Saccharomyces cerevisiae*, we recently developed CalMorph, a high-throughput, high-resolution, image-processing program that allows us to analyze and quantitate 501 cell morphology parameters from fluorescent microscopic images of triple-stained (cell wall, actin, and nuclear DNA) yeast cells [8]. Using CalMorph, we phenotyped more than 200 cells for each of 4718 nonessential gene deletions [8]. Our results revealed that deletions of functionally related genes caused similar morphologic phenotypes, enabling loci to be functionally assigned to a specific cellular pathway [8]. Further detailed phenotypic analysis revealed that calcium treatment induced various morphologic changes in calcium-sensitive *cls* mutants, and that functionally related *cls* mutants could be grouped based on similarities in the calcium-induced phenotypes [9]. These results suggest that the cellular pathways affected by a given reagent can be preliminarily identified based on phenotypic similarities induced by that reagent. Based on these observations, we hypothesized that genetic targets can be inferred using multiparameter comparisons of drug- and mutation-induced morphologic changes.

Here we present a proof-of-concept study that employed four well-characterized bioactive compounds. We developed a Java-based program that uses an inference algorithm to estimate similarities between induced morphologic changes. Using this algorithm to examine 4718 nonessential gene deletion mutants, the previous known target genes of the compounds and the functionally related genes to these targets were successfully identified and potentially affected cellular pathways were revealed, demonstrating the validity of this approach.

## Results

### A high-content image-profiling method

We assumed that dose-dependent morphologic changes induced by a chemical compound would resemble the effects of mutations in genes encoding targets of the compound. Therefore, to infer the targets of potential drugs, we established a high-content, image-profiling procedure. First, to minimize side effects caused by high concentrations of the chemicals, the maximum treatment concentration of each chemical compound was defined as the concentration that produced a slight delay in the growth rate of wild-type yeast cells (approximately 10% of control samples). Three lower concentrations were then selected and wild-type yeast cells were treated with or without the chemical compound at the various concentrations. Wild-type yeast cells grown in the presence of each concentration were fixed and stained with fluorescein isothiocyanate-conjugated concanavalin A (FITC-ConA) to detect the cell wall component mannoprotein, rhodamine-phalloidin (Rh-ph) to detect the actin cytoskeleton, and 4′,6-diamidino-2-phenylindole (DAPI) to detect nuclear DNA. Samples from five independent cultures grown in the presence of each concentration (25 samples for each chemical compound  =  five concentrations × five replications) were examined using the image-processing program CalMorph as described previously [8]. At least 200 cells from each sample were analyzed for 501 morphologic parameters (see Materials and Methods).

**Figure 1:**
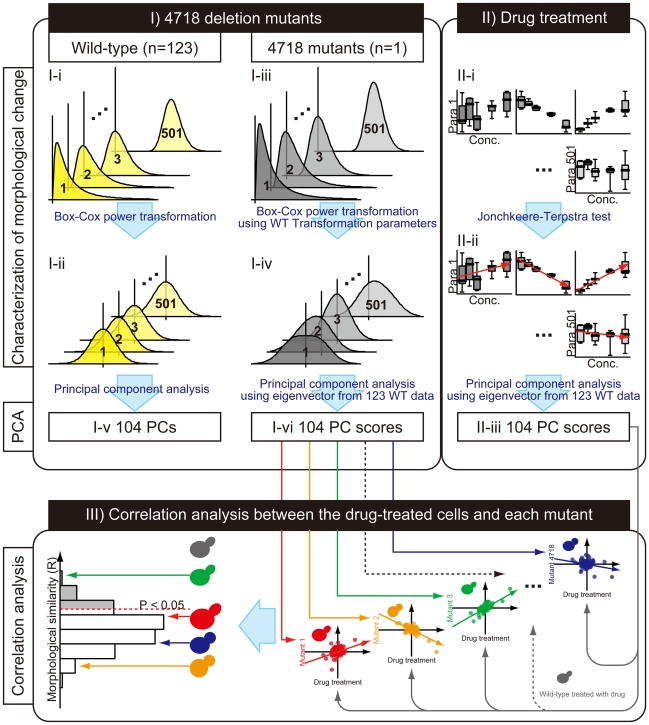
Schematic of the high-content, image-profiling method used in this study. Yellow histograms in I-i and I-ii indicate the original and transformed distributions of 123 wild-type values for each parameter, respectively. Gray histograms in I -iii and I-iv indicate the original and transformed distributions of parameter values for the 4718 mutants, respectively. In I-i, I-ii, I-iii, and I-iv, the vertical and the horizontal histogram axes represent the frequencies and parameter values, respectively. Box plots in II-i and II-ii indicate the distributions of the parameter values from drug-treated wild-type yeast. In the box plots in II-i and II-ii, the vertical and the horizontal axes represent the parameter values and drug concentrations, respectively. Red arrows in II-ii indicate the direction of trends within the parameter data. PC in I-v, I-vi and II-iii indicates the principal components defined by PCA of transformed wild-type data for 501 parameters. The vertical and the horizontal axes in the two-dimensional plots in III indicate the principal component scores for the mutants and drug-treated wild-type cells, respectively. The histogram in III shows the distribution of morphologic similarities between the dose-dependent changes induced drug treatment and those induced by the deletion mutations. Morphologic similarities were defined using the Pearson product-moment correlation coefficient (R); mutants with significantly high positive R values (the gray region in the histogram) were defined as potential targets at two sided *P* <0.05 with the Bonferroni correction, which was calculated based on *P* values from the correlation coefficient test.

The targets of the chemical compounds were inferred using the following three steps: I) characterization and principal component analysis (PCA) of the 4718 deletion mutants; II) characterization and PCA of wild-type cells treated with the chemical compound; and III) correlation analysis of the compound-treated and mutant cells (Figure 1).

To evaluate the 501 parameters in each mutant, the distributions of each parameter value from the 4718 mutants were normalized using a Box-Cox power transformation [10]. Parameters for the transformation were estimated from the wild-type distribution (n = 123; Figure 1 I-i and -ii) using a previously published procedure [8]. Each transformed parameter value for a mutant represented an abnormality relative to the standard normal distribution (Figure 1 I-iii and -iv). Next, the 501 morphologic parameters were summarized with PCA because some of the parameters strongly correlated with each other [8]. We extracted 104 independent axes (principal components) from the 501 wild-type parameter values (n = 123) using PCA at a cumulative contribution ratio greater than 0.99 (Figure 1 I-v). Based on the eigenvector calculated from the wild-type data, the principal component scores for the 104 axes were calculated in each mutant (Figure 1 I-vi). The scores were then used to represent an altered morphologic profile that was associated with deletion of the gene.

To evaluate the dose-dependency of the 501 parameters in chemically treated wild-type cells, 25 sample values for each parameter were summarized into a Z score from the Jonckheere-Terpstra test (Figure 1 II-i and -ii) [11]; this nonparametric statistical test examines ordered differences among classes (*e.g.*, concentrations of the compound). Each Z score represented the dose-dependency of the parameter under a normal distribution. Then, the Z scores for the 501 parameters were mapped with the principal component scores on the 104 axes using the calculated eigenvector from I-v (Figure 1 II-iii). These scores represented the altered morphologic profile that resulted from treatment with the compound.

To evaluate similarities between morphologic changes in drug-treated wild-type cells and mutant strains, we calculated the Pearson product-moment correlation coefficient R and the associated *P* value for the 104 principal component scores from the two samples (Figure 1 III). To detect significant similarity, we estimated two-sided P values by t-test for the correlation coefficient, and set α level at 0.05 with the Bonferroni correction dividing α by 4718.

We developed a Java-based program to perform the statistical estimation, and employed four well-characterized chemical-compounds (hydroxyurea, concanamycin A, lovastatin, and echinocandin B) to evaluate our proposed method (Table 1).

**Table 1:** Chemical compounds used in this study.

Compound	Concentrations	Target cellular process	Target genes
Hydroxyurea	0, 5.0, 10, 20 and 30 mM	Deoxynucleotide triphosphate synthesis	*RNR1*, *RNR2*, *RNR3* and *RNR4*
Concanamycin A	0, 2.0, 3.9, 7.8 and 15 µM	Vacuolar acidification	*VMA1*, *VMA2*, *VMA3*, *VMA4*, *VMA5*, *VMA6*, *VMA7*, *VMA8*, *VMA9*, *VMA10*, *VMA11*, *VMA13*, *VMA16*, *VPH1* and *STV1*
Lovastatin	0, 6.25, 12.5, 25 and 50 µg/ml	Mevalonate synthesis	*HMG1* and *HMG2*
Echinocandin B	0, 0.5, 1, 2 and 3 µg/ml	1,3-beta-glucan synthesis	*FKS1* and *FKS2*

### Hydroxyurea

We used hydroxyurea as a representative of compounds that affect DNA metabolism. Hydroxyurea is used as an antitumor agent with antileukemic activity, which results from inhibition of ribonucleotide reductase activity and consequent suppression of DNA synthesis [12]. The ribonucleotide reductases are α_2_β_2_ tetramers of which structure is highly conserved from bacteria to mammal [13]. The α subunit catalyses reduction of ribonucleotide (Rnr1p and Rnr3p in yeast), whereas the β subunit (Rnr2p and Rnr4p in yeast) producing radicals required for reductase activity of α subunit [14]. The effect of hydroxyurea which quenches the enzyme's tyrosyl radical is specific for radical-producing β subunit [15], [16]. The essential *RNR2* gene was not evaluated in this study because the *rnr2* mutant was not included among the tested 4718 mutants.

**Figure 2:**
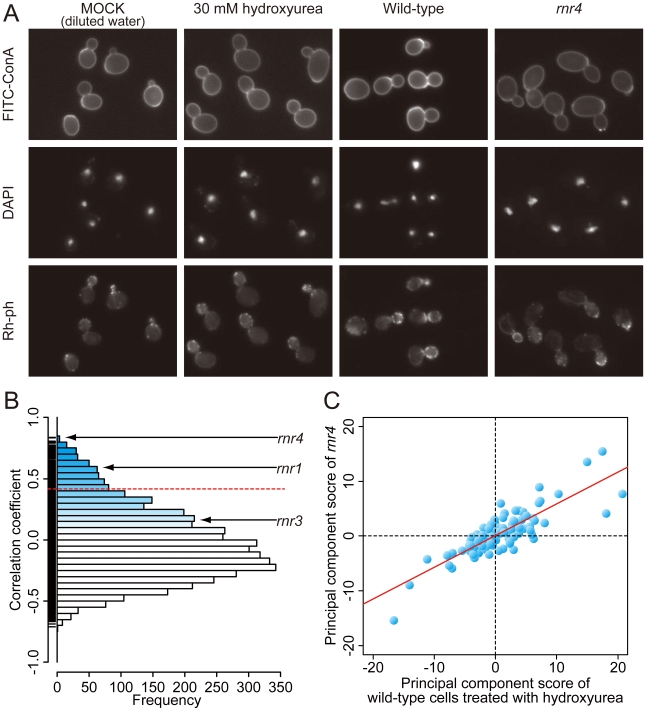
Hydroxyurea treatment and the results of target inference. A) Photographs of wild-type cells treated with 30 mM hydroxyurea and the *rnr4* mutant. Cells were triply stained with FITC-ConA, Rh-ph, and DAPI. Quantitative analysis of the hydroxyurea-induced morphologic changes is summarized in Table S1. B) A histogram of correlation coefficients (R) between the mutant and hydroxyurea-treated wild-type cells. The dashed red line indicates the position of the R value corresponding to the two sided *P* value of 0.05 with the Bonferroni correction based on a correlation coefficient test. C) Two-dimensional plot of wild-type cells treated with hydroxyurea and the *rnr4* mutant. The scores for the 104 principal components are plotted. The red line indicates a linear regression model (R  = 0.738).

Wild-type cells were treated with each concentration of hydroxyurea (Table 1) and photographed (Figure 2A). Images were analyzed using CalMorph and 501 morphologic parameter values were obtained. Using the Jonckheere-Terpstra test, 176, 249, 302, and 366 of the 501 parameters were found to show dose-dependent changes at false discovery rate (FDR)  = 0.01, 0.05, 0.10, and 0.20, respectively [11], [17]. The dose-dependent changes in the 176 parameters (Table S1) indicated that the hydroxyurea-treated wild-type yeast cells were enlarged, with delocalized actin patches in buds and numerous mononuclear budding cells, all of which are typical phenotypes of cells arrested at the S phase of the cell cycle.

To identify mutant cells with similar morphologic profiles, we applied the high-content image profiling on the phenotypic information of the mutants (Figure 1). Among the 4718 mutants, 384 (8.14%) morphologic profiles were significantly similar with that of the hydroxyurea-treated cells by the high-content image-profiling at two sided *P*<0.05 with the Bonferroni correction (Figure 2B). Among the 100 top-ranked mutants (R>0.629), 12 mutants, including a *rnr4* mutant, were categorized as belonging to a “DNA replication” in the gene ontology (GO) database (GOID: 6260). Enrichment for this GO term was significant by GO term finder at *P*<0.05 (Table S2) [18], [19], demonstrating that high-content image profiling efficiently identified genes from pathways related to the function of Rnr4p. Among the three ribonucleotide reductase mutants in the mutant panel, the *rnr1* (R  = 0.592) and *rnr4* (R  = 0.836) mutants were identified based on similar morphologic phenotypes in these mutants and hydroxyurea-treated wild-type yeast (Figure 2B and C, Table 2). Interestingly, among 4718 mutants, disruption of *rnr4*, which encodes the target of hydroxyurea, was associated with the highest R value (Figure 2B).

**Table 2:** Summary of the inference results.

	Number of detected candidates (FDR = 0.01)	Representative target	R value of the representative target mutant	Rank of the target mutant
Hydroxyurea	384 (8.14%)	*rnr4*	0.836 (P<2.20E-16)	1^st^
Concanamycin A	221 (4.68%)	*vma6*	0.685 (P = 1.11E-15)	4^th^
Lovastatin	100 (2.12%)	*hmg1*	0.520 (P = 1.55E-8)	20^th^
Echinocandin B	197 (4.18%)	*fks1*	0.284 (P = 3.51E-3)	527^th^

To investigate whether the utility of high-content image profiling is related to the strength of the mutant phenotypes, we examined the number of parameters affected by deletion of genes encoding various targets of hydroxyurea (*rnr1*, *rnr3*, and *rnr4*). Of the 254 parameters with distributions that could be transformed into a normal distribution [8], 1, 53, and 62 parameters from the *rnr3*, *rnr1*, and *rnr4* mutants, respectively, were significantly different (*P* <0.0001) on one side of the wild-type distribution (Figure 2A), indicating that the *rnr4* and *rnr1* mutants had strong phenotypes relative to the *rnr3* phenotype. The weak phenotype of the *rnr3* mutant, which was not identified as a potential hydroxyurea target, suggested that Rnr3p plays only a minor role in these cellular processes, and that our high-content image-profiling method does not detect weak morphologic phenotypes associated with minor cellular activities.

### Concanamycin A

We used concanamycin A as a representative of compounds that affect intracellular cation homeostasis. Concanamycin A inhibits the proliferation of mouse splenic lymphocytes stimulated by concanavalin A [20], and has been shown to be a specific inhibitor of vacuolar proton-translocating ATPases (V-ATPases) [21]. V-ATPase, a heteromultimeric enzyme consisting of at least 15 subunits (Vma1p-Vma11p, Vma13p, Vma16p, Vph1p, and Stv1p), uses ATP hydrolysis to transport cytosolic protons into vacuoles, resulting in acidification of these compartments [22]. V-ATPase consists of two multimeric subunits; one is hydrophilic V_1_ subunit consisting of 8 components, and the other is hydrophobic V_O_ subunit consisting of 7 components [22]. The photo activated concanamycin A analog binds to subunit c in V_O_ domain from *Manduca sexta* [23], and also in yeast, the binding is specific to subunit c (among c, c′ and c″ subtypes) encoded by *VMA3* in yeast [24]. Because the *vma9* and *vma10* mutants were not included in the mutant panel, we assessed deletions of genes encoding the other 13 V-ATPase subunits.

**Figure 3:**
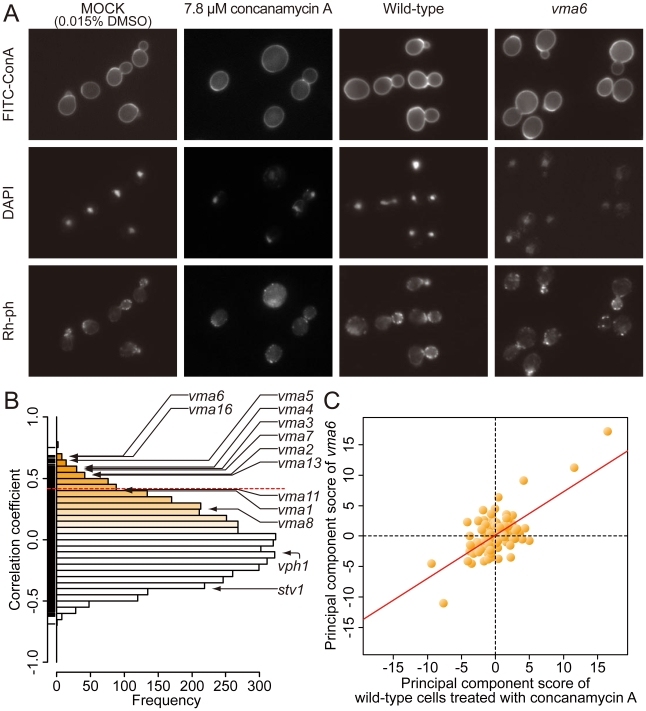
Concanamycin A treatment and the results of target inference. A) Photographs of wild-type cells treated with 7.8 µM concanamycin A and the *vma6* mutant. Cells were triply stained with FITC-ConA, Rh-ph, and DAPI. Quantitative analysis of concanamycin A-induced morphologic changes is summarized in Table S2. B) A histogram of correlation coefficients (R) between mutant and concanamycin A-treated wild-type cells. The dashed red line indicates the position of the R value corresponding to the two sided *P* value of 0.05 with the Bonferroni correction based on a correlation coefficient test. C) Two-dimensional plot of concanamycin A-treated wild-type cells and the *vma6* mutant. Scores for the 104 principal components are plotted. The red line represents a linear regression model (R = 0.685).

Wild-type cells were treated with various concentrations of concanamycin A (Table 1), and examined using the same method described above (Figure 3A). Among the 501 parameters, 0, 54, 111, and 217 parameters showed significantly dose-dependent effects based on the Jonckheere-Terpstra test at FDR  = 0.01, 0.05, 0.10, and 0.20, respectively [11], [17]. Overall, changes in the 54 most sensitive parameters (Table S3) indicated that concanamycin A treatment resulted in rounder cells with bigger nuclei, and fewer mononuclear budded cells were present, all of which are indicative of defective vacuolar acidification.

High-content image profiling revealed that among the 4718 mutants, 221 (4.68%) were significantly similar to concanamycin A-treated cells at two sided *P*<0.05 with the Bonferroni correction (Figure 3B). Among the 100 most likely candidates (R>0.497), nine mutants including *vma3* were associated with “vacuolar acidification” (GOID: 7035), which represented a significant enrichment of this GO term (*P*<0.05, Table S4) [18], [19]. Thus, our method efficiently identified genes encoding proteins in pathways related to V-ATPase. The 8 of 13 V-ATPase mutants were identified (Figure 3B). The maximum R value for the 13 V-ATPase mutants was 0.685 for the *vma6* mutant (Figure 3C). These results suggested that the morphologic features of the 8 mutants were similar to the dose-dependent morphologic changes induced by concanamycin A. Moreover, the 8 V-ATPase mutants among the 221 candidates represented a significant enrichment from the original sample of 13 V-ATPase mutants in the complete panel of 4718 mutants (*P* = 2.45E-7; one-side binomial test).

Because Vma1p, Vma8p, Vma11p, Vph1p and Stv1p do not directly interact with the drugs [23], [24], [25], it is not surprising that they were failed to be identified as the candidates. However, other V-ATPase mutants were able to be detected even if they do not directly interect, suggesting that the functional relationships of the drug target with these components is potentially detected. Then, we reasoned that the undetected V-ATPase mutants show too weak phenotypes. To test the effects of the strength of observed mutant phenotype, we compared the numbers of parameters that were changed by the various V-ATPase gene deletions. Among the 254 parameters with distributions that could be transformed into a normal distribution [8], 32, 23, 21, 15, 10, 8, 6, 3, and 2 parameters were identified with a normal distribution (*P*<0.0001) on one side from the *vma6*, *vma7*, *vma5*, *vma4*, *vma3*, *vma2*, *vma16*, *vma1*, and *vma11* mutants, respectively, whereas no parameters were detected for the *vma8*, *vma13*, *vph1*, and *stv1* mutants. Therefore, the *vma1, vma8, vma11, vph1* and *stv1* were not detected as candidate targets because of the mutants' weak phenotypes.

### Lovastatin

We used lovastatin as a representative of compounds that affect lipid metabolism. Lovastatin is a cholesterol-lowering agent that disrupts cholesterol synthesis by specifically inhibiting hydroxymethylglutaryl-coenzyme A (HMG-CoA) reductase in the mevalonate pathway [26]. HMG-CoA reductases encoded by *HMG1* and *HMG2* in yeast are the rate-limiting enzyme in the sterol biosynthetic pathway [27].

**Figure 4:**
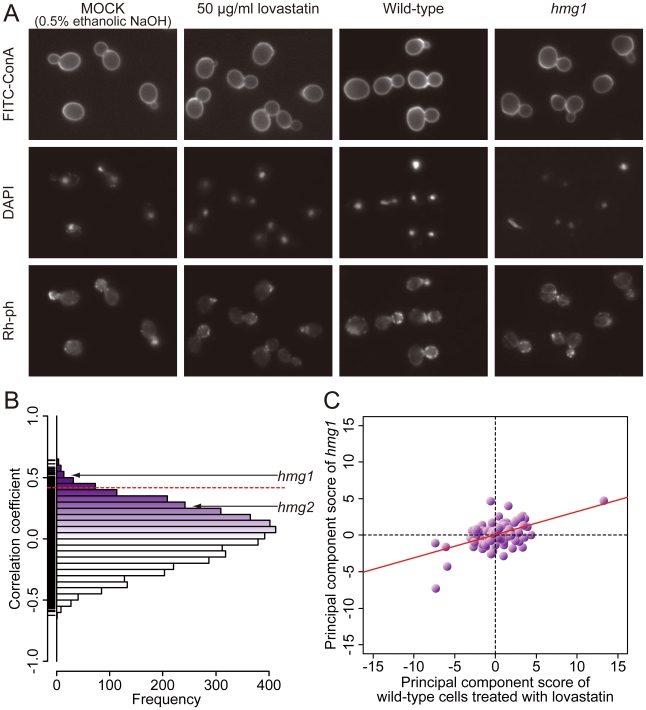
Lovastatin treatment and the results of target inference. A) Photographs of wild-type cells treated with 50 µg/ml lovastatin and the *hmg1* mutant. Cells were triply stained with FITC-ConA, Rh-ph, and DAPI. Quantitative analysis of lovastatin-induced morphologic changes is summarized in Table S3. B) A histogram of correlation coefficients (R) between the mutant and lovastatin-treated wild-type cells. The dashed red line indicates the position of the R value corresponding to the two sided *P* value of 0.05 with the Bonferroni correction based on a correlation coefficient test. C) Two-dimensional plot of lovastatin-treated wild-type cells and the *hmg1* mutant. Scores for the 104 principal components are plotted. The red line represents a linear regression model (R = 0.512).

Wild-type cells were treated with various concentrations of lovastatin (Table 1) and the cells were characterized using CalMorph (Figure 4). Jonckheere-Terpstra tests at FDR  = 0.20 identified significant dose-dependent effects for 56 of the 501 parameters [11], [17]. Overall, the changes indicated that lovastatin treatment made wild-type yeast cells smaller and rounder, and decreased the percentage of large bud cells (Table S5).

High-content image profiling detected 100 (2.12%) of the 4718 genes as potential lovastatin targets at two sided *P*<0.05 with the Bonferroni correction (Figure 4B). Three of the mutated genes, including *fen2*, were associated with “vitamin transporter activity” (GOID: 51183), a GO term that was significantly enriched (*P*<0.05, Table S6) among the top 100 candidate targets (R>0.417). Fen2p is a plasma membrane H^+^-pantothenate symporter [28] and CoA is synthesized from pantothenate [29], demonstrating that our high-content image-profiling method identified genes from pathways related to the mevalonate pathway. Although the *hmg2* mutant was not detected as a candidate, the mutant of the lovastatin target *hmg1* was identified (R = 0.520; Figure 4B and C).

Previous results [8] detect no more than two parameters from the *hmg1* and *hmg2* mutants with normal distributions on one side at *P*<0.0001, suggesting that our high-content image-profiling method can detect mutants of genes with major cellular roles even if the mutant show a weak phenotype.

### Echinocandin B

We used echinocandin B as representative of agents that damage the cell wall. Echinocandin B, which is produced by *Aspergillus nidulans* and *Aspergillus rugulosus* [30], has an antifungal activity owing to an specific inhibition of the synthesis of 1,3-β-D-glucan, a major component of the yeast cell wall [31]. Because both Fks1p and Fks2p have been identified as the catalytic subunit of 1,3-β-D-glucan synthase and the synthase activity of a *fks1* mutant was reported to be significantly lower than those of wild-type cells and a *fks2* mutant [32], [33], we believe Fks1p is a representative target of echinocandin B.

Using Jonckheere-Terpstra tests at FDR  = 0.01, 226 of 501 parameters showed significant dose-dependency in response to echinocandin B treatment (Table S7 and Table 1). The echinocandin B-treated wild-type yeast cells were larger and rounder with bigger nuclei and necks. The cells contained delocalized actin and fewer large buds were observed.

Among the 4718 mutants, 197 (4.18%), were detected as candidates using high-content image profiling at two sided *P*<0.05 with the Bonferroni correction. In the top 100 candidates, three mutants that were categorized as having “α-1,6-mannosyltranserase activity” (GOID: 9) were identified, which was a significant enrichment of this GO term (*P*<0.05, Table S8) [18], [19]. The results demonstrate that our high-content image-profiling method efficiently identified genes from pathways related to cell wall biosynthesis, although neither *fks1* nor *fks2* mutant were included in the candidates.

Based on previous results [8], 10 of the 254 parameters for the *fks1* mutant showed normal distributions on one side (*P*<0.0001), whereas no parameters were detected for the *fks2* mutant Since, it has been suggested that Fks1p has other functions than 1,3-β-glucan synthesis, a combination of the redundancy and the multifunctionality might make it difficult to detect the targets, even if the phenotype is not so weak [34], [35].

## Discussion

We developed a high-content image-profiling system to identify drug targets systematically. The multiparameter profiling method uses 501 morphologic parameters and a dataset of 4718 nonessential deletion mutants (Figure 1). We used four well-characterized compounds that affect various cellular functions as test cases (Table 1). The drug target candidates were successfully screened into 2% to 8% from 4718 mutants on the panel, and the previously reported targets of the compounds and gene products associated with pathways that were functionally related to the target were enriched among the candidates. These results indicate that our high-content image-profiling method can detect targets of drug candidate and uncover their potential mechanisms of action.

The proposed method allows systematic, high-content screening independent of previous information about the drug candidate. The systematic approach with defined statistical criteria means that this method can theoretically be used not only to identify candidates of the drug targets but also to assess the effects of any condition that induces morphologic changes (*e.g*., genetic mutations, nutritional starvation, and temperature shifts, among others). Further, because the analysis is statistics-based, the methodology can be expanded to higher eukaryotes if a morphologic phenotype database is available. The statistical power and utility of the method are enhanced by the multiple parameters that are extracted from high-resolution images. Examination of multiple intracellular activities and detailed phenotypic information for each mutant allow assessments of the effects of a drug on a wide variety of cellular functions. Our high-content image-profiling method also allows direct and systematic identification of drug targets from 4718 nonessential genes without any prior knowledge of about the mechanism of action of the candidate drug. This contrasts with conventional high-content imaging approaches, which focus on specific bioactivities (*e.g.*, translocation of fluorescently labeled cellular targets between intracellular compartments) to assign the drug candidate into well-characterized groups [36], [37]. In addition, the off-target effects may be estimated by comparing results from low concentration of the drug treatment (reflecting target-specific effect) with that from high concentration (reflecting non-specific effects).

Compendium approaches that have been used with microarray technology to identify genetic targets comprehensively and systematically [1], [38] are similar approach to ours. In the compendium approaches, multiparametric profiles similar to that under the query conditions are surveyed from a collection of profiles under various conditions (drug, deletion mutants and etc). The profiles based on the relative gene expression abundance (∼300 profiles of ∼6000 genes) [5], the synthetic lethality (∼1700 profiles of ∼3900 genes) [39] and the fitness (∼80 profiles of ∼3400 genes) [6] are used as compendium data set. These compendium approaches used hydroxyurea as a test case to assess feasibility of target inference, and their results were consistent with that of this study ([5], [6], [39] and data not shown). These approaches and our high-content method may be complementary for target identification because of different screening criteria (*i.e*., fitness versus morphology). Moreover, our approach may be particularly useful to identify targets of drugs that have no apparent effect on cellular fitness.

A key point for our high-content image-profiling is to detect the morphological changes. Therefore, if there are no or very little morphologic changes, it becomes difficult to identify drug targets. As expected, candidates with minor morphologic changes could not be detected. For example, when the drug target is the gene product possessing functionally redundant proteins (e.g., *VPH1* and *STV1* [40]) and/or is associated with relatively lower enzymatic activity (e.g., Rnr3p [41], Hmg2p [27], and Fks2p [32]). In addition, in the case of a multifunctional protein (e.g., Fks1p [34], [35]), detection of the target was difficult even with marked morphologic changes [8]. Nevertheless, as Hmg1p, which showed almost no morphologic change [8] was identified as the target candidate of lovastatin, even with very weak phenotype it may be possible to identify the candidate, suggesting that strong statistical power is needed to detect target-related mutants which show weak phenotypes (e.g., *vma1*, *vma8* and *vma11*). In order to better the statistical power, improvement in the reference data set of deletion mutants (e.g., preparation of the replicated data sets) would be required.

The current version of our high-content image-profiling system is limited to nonessential genes because morphologic phenotypes of mutants carrying deletions in essential genes are not available. Some genetic techniques may allow us to overcome this limitation. For example, heterozygous deletion mutants in diploid yeast may cause a haploinsufficient phenotype and enable essential genes to be screened [2]. Alternatively, a comprehensive set of temperature-sensitive mutants for essential genes [42], including multiple mutant alleles of the same gene, may provide intragenic insights into the inhibitory mechanisms of compounds even if the target genes are multifunctional (e.g., *FKS1* [34], [35]). Finally, morphologic information from overexpression mutants may facilitate the identification of functionally promoted genetic targets [43], [44].

To further improve our method, we can examine more image parameters from of other organelles. The new version of CalMorph can be used to examine 1111 morphologic parameters, including additional characteristics of various cellular components such as mitochondria, vacuoles and spindle pole bodies [45]. The new version of CalMorph should improve the inference accuracy and generality of this approach for a variety of chemicals by expanding the available morphologic data from the mutant panel.

## Materials and Methods

### Media and strain

*S. cerevisiae* were grown in rich medium consisting of YPD medium, 1% (w/v) Bacto yeast extract (BD Biosciences, CA, USA), 2% (w/v) Bacto peptone (BD Biosciences), and 2% (w/v) glucose. The wild-type *S. cerevisiae* strain (access number: Y02458: *MATa his3::KanMX leu2 met15 ura3*) used in this study was purchased from the European *Saccharomyces cerevisiae* Archive for Functional Analysis (EUROSCARF: http://web.uni-frank furt.de/fb15/mikro/euroscarf/).

### Chemicals and test conditions

Stock solutions of 2 M hydroxyurea (Sigma-Aldrich, MO, USA), 100 mM concanamycin A (Sigma-Aldrich), 20 mg/ml lovastatin (Wako Pure Chemical Industries, Osaka, Japan), and 2 mg/ml echinocandin B (gift from Dr. T. Watanabe) were prepared in various solvents: distilled water (DW); dimethyl sulfoxide (DMSO) (Wako Pure Chemical Industries, Ltd.); ethanolic NaOH containing 15% (v/v) ethanol (Wako Pure Chemical Industries) and 0.25% (w/v) NaOH (Wako Pure Chemical Industries) in DW; and DMSO, respectively.

To grow wild-type yeast treated with each reagent, cells were precultured in rich medium, and were used to inoculate wells of a microtiter plate containing fresh rich medium and various inhibitory concentrations of the reagent or the solvent alone (flat-bottomed 96-well plates with lids; Asahi Techno Glass Corporation, Chiba, Japan). The cultures were incubated at 25°C in a shaking incubator (Micro mixer E-36, Taitec Corporation, Saitama, Japan) and their optical densities were measured at 600 nm at more than 10 time points over the course of 2 days using a SpectraMax Plus^384^ spectrophotometer (Molecular Devices, CA, USA). Growth tests were performed twice for each reagent, the averaged doubling time was calculated, and the concentration that delayed the doubling time by approximately 10% was determined.

### Image acquisition and analysis

Cells (8×10^6^ cells/ml) growing at 25°C in rich medium with or without various concentrations of each reagent (Table 1) were fixed in medium supplemented with 3.7% formaldehyde (Wako Pure Chemical Industries) and 0.1 M potassium phosphate buffer (pH 6.5). Triple staining of the yeast cells and image analysis with CalMorph (ver. 1.1) were performed as described previously [8]. CalMorph automatically characterizes each yeast cell by calculating 501 morphologic parameters based on data from more than 200 cells. Five independent cultures grown under each condition were analyzed.

### Statistical analysis

Calculation of the doubling time and plotting of the analyzed data was performed using R (http://www.r-project.org/). High-content image-profiling, including the PCA, Jonckheere-Terpstra test, Pearson product-moment correlation analysis, and bootstrap-based estimation of the FDR [17], was performed using a newly built Java program. The Java program includes a graphical user interface and is available upon request. Morphologic data about the 4718 mutants and 126 replications of wild-type cells were described previously [8]. Among the replicated wild-type data, three replications were discarded because of missing values. A Box-Cox power transformation was used as described previously [8]. GO::TermFinder, a set of Perl modules, was downloaded from http://search.cpan.org/dist/GO-TermFinder/and used to examine enrichment of GO terms in 4718 genes [19]. In order to implement GO::TermFinder at local computer, two files including gene information and ontology information were downloaded from the *Sacchromyces* Genome Database (http://www.yeastgenome.org/) [18] and the Gene Ontology website (http://www.geneontology.org/) [46], respectively.

## Supporting Information

Table S1 A list of parameters identified from hydroxyurea-treated cells using Jonckheere-Terpstra tests at FDR  = 0.01. Green, red, and blue in the ‘ID’ column indicate parameters that were calculated from images of the cell wall, actin, and DNA, respectively. Red and green in the ‘P value’ column indicate increases and decreases, respectively, in the parameter values following hydroxyurea treatment with a given FDR from the Jonckheere-Terpstra test. All 501 parameters were described previously. A) A list of parameters detected at stage A (unbudded cells with one nucleus). B) A list of parameters detected at stage A1B (budded cells with one nucleus). C) A list of parameters detected at stage C (budded cells with two nuclei). D) A list of parameters representing various cellular ratios.
(0.06 MB XLS)

Table S2 A list of GO terms enriched in the top 100 genes ranked by the high-content image-based profiling for hydroxyurea treatment. Of the 4718 genes, 4379 genes were associated to at least one GO term, in which 91 of top 100 genes ranked by high-content image-profiling for hydroxyurea were included. Using the GO term finder (see Materials and Methods), significant enrichment of 111 GO terms was detected at P<0.05 with the Bonferroni correction (Corrected P-value column) among the 91 genes from 4379 genes, and GO terms associated with 100 and fewer genes in the 4379 genes (Number of annotations in the 4718 genes column) were listed.
(0.03 MB XLS)

Table S3 A list of parameters identified from concanamycin A-treated cells using Jonckheere-Terpstra tests at FDR  = 0.05. The color scheme is the same as that used in Table S1. A) A list of parameters detected at stage A (unbudded cells with one nucleus). B) A list of parameters detected at stage A1B (budded cells with one nucleus). C) A list of parameters detected at stage C (budded cells with two nuclei). D) A list of parameters that represent various cell ratios.
(0.04 MB XLS)

Table S4 A list of GO terms enriched in the top 100 genes ranked by the high-content image-based profiling for concanamycin A treatment. Of the 4718 genes, 4379 genes were associated to at least one GO term, in which 90 of top 100 genes ranked by high-content image-profiling for concanamycin A were included. Using the GO term finder (see Materials and Methods), significant enrichment of 89 GO terms was detected at P<0.05 with the Bonferroni correction (Corrected P-value column) among the 90 genes from 4379 genes, and GO terms associated with 100 and fewer genes in the 4379 genes (Number of annotations in the 4718 genes column) were listed.
(0.03 MB XLS)

Table S5 A list of parameters identified from lovastatin-treated cells using Jonckheere-Terpstra tests at FDR  = 0.20. The color scheme is the same as Table S1. A) A list of parameters detected at stage A (unbudded cells with one nucleus). B) A list of parameters detected at stage A1B (budded cells with one nucleus). C) A list of parameters detected at stage C (budded cells with two nuclei). D) A list of parameters that represent various cell ratios.
(0.04 MB XLS)

Table S6 A list of GO terms enriched in the top 100 genes ranked by the high-content image-based profiling for lovastatin treatment. Of the 4718 genes, 4379 genes were associated to at least one GO term, in which 92 of top 100 genes ranked by high-content image-profiling for lovastatin were included. Using the GO term finder (see Materials and Methods), significant enrichment of three GO terms was detected at P<0.05 with the Bonferroni correction (Corrected P-value column) among the 92 genes from 4379 genes, and GO terms associated with 100 and fewer genes in the 4379 genes (Number of annotations in the 4718 genes column) were listed.
(0.02 MB XLS)

Table S7 A list of parameters identified from echinocandin B-treated cells using Jonckheere-Terpstra tests at FDR  = 0.01. The color scheme is the same as Table S1. A) A list of parameters detected at stage A (unbudded cells with one nucleus). B) A list of parameters detected at stage A1B (budded cells with one nucleus). C) A list of parameters detected at stage C (budded cells with two nuclei). D) A list of parameters that represent various cell ratios.
(0.06 MB XLS)

Table S8 A list of GO terms enriched in the top 100 genes ranked by the high-content image-based profiling for echinocandin B treatment. Of the 4718 genes, 4379 genes were associated to at least one GO term, in which 84 of top 100 genes ranked by high-content image-profiling for echinocandin B were included. Using the GO term finder (see Materials and Methods), significant enrichment of 52 GO terms was detected at P<0.05 with the Bonferroni correction (Corrected P-value column) among the 84 genes from 4379 genes, and GO terms associated with 100 and fewer genes in the 4379 genes (Number of annotations in the 4718 genes column) were listed.
(0.03 MB XLS)
